# PPARβ/δ: Linking Metabolism to Regeneration

**DOI:** 10.3390/ijms19072013

**Published:** 2018-07-10

**Authors:** Ajit Magadum, Felix B. Engel

**Affiliations:** 1 Cardiovascular Research Center, Icahn School of Medicine at Mount Sinai, New York, NY 10029, USA; ajit23882@gmail.com; 2 Department of Genetics and Genomic Sciences, Icahn School of Medicine at Mount Sinai, New York, NY 10029, USA; 3 Department of Nephropathology, Experimental Renal and Cardiovascular Research, Institute of Pathology, Friedrich-Alexander-Universität Erlangen-Nürnberg, 91054 Erlangen, Germany; 4 Muscle Research Center Erlangen (MURCE), 91054 Erlangen, Germany

**Keywords:** PPARβ/δ, regeneration, proliferation, differentiation, metabolism, Wnt signaling, PDK1, Akt, glycolysis

## Abstract

In contrast to the general belief that regeneration is a rare event, mainly occurring in simple organisms, the ability of regeneration is widely distributed in the animal kingdom. Yet, the efficiency and extent of regeneration varies greatly. Humans can recover from blood loss as well as damage to tissues like bone and liver. Yet damage to the heart and brain cannot be reversed, resulting in scaring. Thus, there is a great interest in understanding the molecular mechanisms of naturally occurring regeneration and to apply this knowledge to repair human organs. During regeneration, injury-activated immune cells induce wound healing, extracellular matrix remodeling, migration, dedifferentiation and/or proliferation with subsequent differentiation of somatic or stem cells. An anti-inflammatory response stops the regenerative process, which ends with tissue remodeling to achieve the original functional state. Notably, many of these processes are associated with enhanced glycolysis. Therefore, peroxisome proliferator-activated receptor (PPAR) β/δ—which is known to be involved for example in lipid catabolism, glucose homeostasis, inflammation, survival, proliferation, differentiation, as well as mammalian regeneration of the skin, bone and liver—appears to be a promising target to promote mammalian regeneration. This review summarizes our current knowledge of PPARβ/δ in processes associated with wound healing and regeneration.

## 1. Introduction

Humankind has been fascinated by the phenomenon of regeneration since ancient history. While the phenomenon of regeneration is already mentioned in Greek mythology (punishment of Prometheus or Hercules’ second labor—slaying the Lernean Hydra), the first written records date back to Empedocles (490–430 BCE) and Aristotle (384–322 BCE, lizard tail regeneration in his books *History of Animals* and *Generations of Animals*). The first known scientific publication appeared in 1712, in which René Antoine Ferchault de Réaumur described limb regeneration in crustaceans [1]. Considering the implications of regeneration, there was and is still great public interest. Initially, the observation of the possibility of the regeneration of entire animals (e.g., hydra (*Hydra vulgaris*) and planaria (*Schmidtea mediterranea*)) resulted in heated philosophical and religious discussions: can the soul be split? Where is its residence? Yet, there was also great optimism. For example, the great philosopher “Voltaire marveled briefly: he saw at once that the loss and replacement of one’s head presented serious problems for those who saw that structure as the seat of a unique “spirit” or soul: and thought of the possible consequences of the experiment for man. Writing at this time to poor blind Madame du Deffand, he lamented that for snails but not for her the replacement of bad eyes by good was a possibility. Later he expressed confidence that men would one day so master the process of regeneration that they too would be able to replace their entire heads. There are many people, he implied, for whom the change could hardly be for the worse” [2]. Yet, while it appears soon possible to transplant a human head [3], we are far away from being able to induce the regeneration of damaged tissues/organs in humans.

In 1901, Thomas Morgan defined “regeneration” as “the replacement of missing structures following injury” [4]. It is often assumed that regeneration includes the restoration of structure and function of lost or damaged organs/tissues. Yet, during mammalian liver regeneration, upon resection of a liver lobe, the function is restored by increasing the size of the remaining lobes, not by re-growing a new lobe [5]. Thus, the main aim in regenerative medicine is to restore tissue/organ function.

The ability of regeneration is widely and randomly distributed in the animal kingdom [6]. Yet, the efficiency and extent of regeneration varies greatly. For example, *Hydra vulgaris* and *Schmidtea mediterranea* are considered immortal as they can reform from an individual, specialized cell type [6]. Amphibians and fish such as the newt *Notophthalmus viridescens* and the zebrafish *Danio rerio* can regenerate a large variety of organs including appendages, heart, lens, retina, and central nervous system [7]. Mammals are more restricted in their regenerative capacity, even though they can for example recover from blood loss as well as damage to the peripheral nervous system, skeletal muscle, and liver. Yet, as humans cannot recover from damage to essential organs such as heart and brain, there is a great interest in understanding the molecular mechanisms of natural occurring healing and regeneration and to apply this knowledge to repair human tissues/organs upon injury.

Peroxisome proliferator-activated receptor (PPAR) β/δ has been demonstrated, as described in detail below, to be involved in several key cellular processes relevant to regeneration: proliferation, differentiation, migration, and apoptosis. In addition, PPARβ/δ plays important roles in metabolism, angiogenesis, and inflammation that have been identified as important processes in regeneration. Thus, the aim of this review is to summarize the potential of PPARβ/δ as a therapeutic target for regenerative therapies.

## 2. PPARβ/δ

Three PPAR isoforms have so far been identified which are designated PPARα, PPARβ/δ, and PPARγ. They belong to the nuclear-receptor superfamily, meaning they act as transcription factors upon ligand activation. PPARβ/δ can be activated by endogenous ligands like polyunsaturated fatty acids and eicosanoid metabolites (e.g., prostacyclin and 15-hydroxyeicosatetraenoic acid (15-HETE)) as well as artificial agonists including GW501516, GW0742, L-165041, and carbacyclin [8,9]. In addition, the action of PPARβ/δ can be inhibited by several inverse agonists and antagonists [10]. Yet, there are currently neither agonistic nor antagonistic drugs clinically available [10,11].

PPARβ/δ is as a nuclear receptor characterized by classical domains: an N-terminal region containing a ligand-independent transactivation domain, often known as activation function 1 (AF-1), a DNA-binding domain (DBD), a flexible hinge region, and an AF-2 domain including a ligand-binding domain (LBD) and a ligand-dependent transactivation domain. The principle mode of action of PPARβ/δ is the heterodimerization with the 9-cis retinoic acid receptor (RXR or NR2B) and binding via two zinc-fingers in the DBD to peroxisome proliferator response elements (PPREs) located in the promoter regions of their target genes [12]. Chromatin immunoprecipitation sequencing has revealed three types of target genes [13]: (i) PPARβ/δ-RXR binds to PPREs as a repressor complex. Expression of such genes is induced upon siRNA-mediated depletion of PPARβ/δ but not by agonists; (ii) Type II genes are regulated as Type I genes but can be activated by agonists (canonical regulation); (iii) The third class of genes contains only PPRE-like motifs. They are bound by PPARβ/δ containing complexes which act as transcriptional activators. Expression of such genes is downregulated upon siRNA-mediated depletion of PPARβ/δ and respond weakly, if at all, to ligands. In addition, PPARβ/δ can regulate transcription independently of DNA binding by suppressing transcription factors via direct physical interaction, competition for limiting amounts of shared co-activators, and inhibition of mitogen-activated protein kinase (MAPK) signaling [12]. For instance, PPARβ/δ inhibits the nuclear factor κ-light-chain-enhancer of activated B cells (NF-κB) pathway by interacting with the NF-κB subunit p65 and thereby decreasing NF-κB binding to the DNA resulting in the inhibition of the transcription of NF-κB target genes [8]. In addition, it has been shown that PPARβ/δ interacts with β-catenin in colon cancer cells controlling the expression of vascular endothelial growth factor (VEGF) A [14]. In cardiomyocytes PPARβ/δ induces via β-catenin Cyclin D2 and c-MYC [9]. Another example is the interaction with the corepressor B-cell lymphoma 6 (BCL-6) [8,13]. It is important to note that PPARβ/δ exhibits different regulatory roles on the same gene dependent on its environment. This dependency on its environment explains the later described cell type-specific roles of PPARβ/δ. For example, it has been reported that several different signaling kinases can modulate the transcriptional activity of PPARβ/δ including protein kinase A and p38 mitogen-activated protein kinase [8]. In addition, PPARβ/δ is widely expressed with relative abundant expression in the brain, skeletal muscle, heart, gut, placenta, and skin [15,16,17,18,19,20]. It has been shown to play major roles in fatty acid metabolism and energy expenditure and thus also in skeletal and cardiac muscle homeostasis and disease as well as metabolic disorders [11,12,21]. In addition, PPARβ/δ is involved in a variety of other diseases such as cancer (including inflammation, cell survival and angiogenesis) [13,22,23], skin disease [24,25,26], atherosclerosis [27], retinopathy [28], and Alzheimer’s disease [29]. Finally, more and more reports suggest that modulation of PPARβ/δ activity might provide an opportunity for wound healing and tissue regeneration [9,25,30,31,32,33,34].

## 3. PPARβ/δ Controls Basic Mechanisms of Wound Healing and Regeneration

Tissue protection, healing, and regeneration require a tight control of several processes including apoptosis (e.g., due to increased functional demand or lack of oxygen), proliferation and/or differentiation of stem cells to generate lost cell types, as well as extracellular matrix remodeling and breakdown (e.g., resolving scar tissue and restoration of a tissue support matrix). The analysis of natural occurring regeneration in model organisms such as zebrafish, newt, and the Murphy Roths Large (MRL) mouse have revealed that healing and regeneration depend on hypoxia-induced signaling, inflammation induced by inflammatory cytokines and eicosanoids produced during the first hours after injury, secretion of pro-angiogenic factors, and metabolic alteration [7,35,36,37,38,39,40]. In the following subchapters, we will highlight the roles of PPARβ/δ in these processes.

### 3.1. Energy Metabolism

In adult mammals, the vast majority of energy is produced by oxidative metabolism in mitochondria. In recent years, it has been proposed that this oxidative metabolism as basal metabolic state underlies the low regenerative capacity in mammals [37]. Accumulating evidence indicates that changes in metabolism, namely the activation of glycolysis, play an important role in regeneration. For example, increased glycolysis is correlated with planarian regeneration [41]. Moreover, inhibition of glycolysis impairs neonatal heart [42] as well as adult skeletal muscle [43] regeneration in mice. Intriguingly, the enhanced regenerative capacity of the MRL mouse has been attributed to increased glycolysis and reduced fatty acid oxidation as their basal metabolic state. Accordingly, enhancing fatty acid oxidation in MRL mice inhibited regeneration [37]. As PPAR plays an important role in energy metabolism [44,45] it is not surprising that PPARβ/δ activity/signaling is required for regeneration (see Section 4). However, even though regeneration is associated with glycolysis it remains elusive what advantage glycolysis has over an oxidative metabolism which generates 18 times as much ATP per mole of glucose. One issue might be that the utilization of oxygen results in reactive oxygen species which can oxidize lipids, nucleic acids, and proteins and thus might result in cellular dysfunction.

In high energy demand tissues (e.g., cardiac and skeletal muscle, brown adipose tissue) PPARβ/δ overexpression increases the expression of genes involved in fatty acid transport and beta-oxidation. Concordantly, PPARβ/δ deletion resulted in decreased expression of these genes [46,47,48,49,50,51]. Furthermore, PPARβ/δ overexpression increases in skeletal muscle the proportion of oxidative fibers [47] while PPARβ/δ deletion markedly increases glycolytic fibers with reduced fatty acid oxidation [52]. In contrast to muscle, PPARβ/δ overexpression in liver increases glucose utilization and lipogenesis [53]. Notably, PPARβ/δ overexpression in the heart does also increase glucose utilization via increased glucose transporter type 4 (Glut4) expression [54]. The principle regulatory mechanism is the protein-protein-interaction of PPARβ/δ with PPAR coactivator 1α (PGC1α) and nuclear receptor corepressor 1 (NCOR1) which are lacking DNA binding activity [45]. The resulting complexes regulate the transcription of genes encoding for example forkhead box protein O1 (FoxO1), pyruvate dehydrogenase kinase 4 (PDK4), cluster of differentiation 36 (CD36), lactate dehydrogenase B, and lipoprotein lipase [21,44].

### 3.2. Apoptosis

The link between apoptosis and metabolism has been known for a long time. One of the best examples of this link is the dual functionality of cytochrome c. On one hand, the metabolic role of cytochrome c is to pass an electron from respiratory complex III to complex IV in order to promote adenosine triphosphate (ATP) generation through oxidative phosphorylation. On the other hand, it is required for apoptosis in order to activate caspases. Notably, the apoptotic function of cytochrome c is inhibited by glucose-stimulated production of intracellular glutathione, a mechanism utilized by glycolytic cancer cells [55]. Considering the role of apoptosis during healing or regeneration usually results in the conclusion that inhibition of apoptosis is beneficial. However, apoptosis can also be beneficial as recently reviewed by Diwanji and Bergmann in the context of apoptosis-induced compensatory proliferation [56].

PPARβ/δ mediates retinoic acid-stimulated keratinocyte survival [57,58,59] and ligand activation of PPARβ/δ inhibited palmitate-induced apoptosis in neonatal cardiomyocytes by preventing an increase in interleukin (IL)-6 levels [60]. Concordantly with an anti-apoptotic function, inhibition of PPARβ/δ upon stimulation with 13-*S*-hydroxyoctadecadienoic acid induces apoptosis in colorectal cancer cells [61]. In addition, PPARβ/δ is required for the VEGF-mediated maintenance of endothelial cell (EC) survival [62]. In contrast, telmisartan (an angiotensin II receptor antagonist; used in the management of hypertension) stimulates in a PPARβ/δ-dependent manner apoptosis in prostate cancer cells [63]. Notably, the PPARβ/δ ligand retinoic acid (RA) can exhibit pro- as well as anti-apoptotic effects. The effect of RA depends on intracellular lipid binding proteins which transport RA to a specific nuclear receptor. RA is pro-apoptotic in cells in which cellular retinoic acid binding protein (CRABP)-II transports RA to the nucleus, mediating interaction with the RA receptor. In contrast, RA is anti-apoptotic in cells in which fatty acid binding protein (FABP) 5 transports RA to the nucleus mediating interaction with PPARβ/δ [57]. Yet, even though it is well known that PPARβ/δ regulates cell survival in several cell types, little is known about downstream signaling pathways. In keratinocytes PPARβ/δ prevented apoptosis by modulating Akt signaling via transcriptional upregulation of integrin-linked kinase (ILK) and PDK1 [58]. Experiments on ECs revealed that PPARβ/δ inhibits apoptosis by binding to the promoter of 14-3-3α resulting in increased 14-3-3α protein levels, reduction of Bad translocation to mitochondria via direct protein–protein interaction, and inhibition of Bad-triggered apoptosis [64]. This anti-apoptotic pathway is shared by all PPARs [65]. In addition, it has been shown that PPARβ/δ inhibits oxidative stress-induced apoptosis in H9c2 cells (rat cardiac myoblast) by a direct transcriptional activation of catalase gene expression [66] as well as in adult rat cardiomyocytes [67].

### 3.3. Inflammation: Fibrosis and/or Regeneration

Injury results in fibrosis/scaring or in healing/regeneration with or without transient fibrosis. Already, decades ago, it was assumed that fibrosis might inhibit endogenous repair mechanisms. Yet, initial attempts to enhance healing/regeneration by inhibiting fibrosis has been shown to be detrimental, for example resulting in heart wall rupture after myocardial infarction [68]. In recent years, accumulating evidence has been provided that fibrosis and regeneration are inversely correlated with each other [69,70]. Thus, great effort is invested to identify novel approaches to modulate scar formation and to promote healing/regeneration at the same time [71,72,73]. The major players in controlling the response to injury are inflammatory monocytes, tissue-resident macrophages, and fibroblasts [72]. Disturbances in macrophage function such as uncontrolled production of inflammatory macrophages or failed communication between macrophages and other cells such as tissue progenitor cells repress endogenous regenerative mechanisms. The importance of these processes has, for example, been demonstrated by utilizing the MRL as well as African spiny mouse. In these regenerative model systems, anti-inflammatory agents or macrophage depletion blocked ear hole closure [37]. Thus, it is assumed that modulations of inflammatory processes together with anti-fibrotic signals are required to promote regeneration.

Several studies have demonstrated that PPARβ/δ has direct anti-fibrotic effects. For example, it has been shown that genetic and/or pharmacological activation of PPARβ/δ decreases fibrosis in a model of corneal damage [74] as well as myocardial infarction [9]. On a cellular level, agonist treatment inhibits keratinocyte transdifferentiation into myofibroblasts and thus extracellular matrix (ECM) synthesis [74]. During cardiac fibrosis, PPARβ/δ is expressed both in cardiac fibroblasts as well as myofibroblasts. PPARβ/δ activation reduced the proliferation of both cell types, myofibroblast differentiation and collagen synthesis [75]. In addition, high-salt diet-induced fibrosis was associated with PPARβ/δ downregulation whereas capsaicin-inhibited fibrosis via the receptor transient receptor potential vanilloid type 1 (TRPV1) was associated with PPARβ/δ upregulation [76].

That inflammation and innate immunity are processes driven by aerobic glycolysis [77,78,79] indicates that modulation of PPARβ/δ activity might allow modulating these processes to promote regeneration. Yet, while PPARs have been identified as key regulators of inflammatory and immune responses, the role of PPARβ/δ in modulating inflammation during regeneration is poorly characterized. The anti-inflammatory properties of PPARβ/δ are mainly based on inhibiting NFκB signaling [8] as well as expression of pro-inflammatory cytokines (inducible nitric oxide synthases (iNOS), cyclooxygenase (COX) 2, tumor necrosis factor (TNF) α, and adhesion molecules (VCAM-1, ICAM-1, and E-selectin) in macrophages [8,12,27,80]. PPARβ/δ can for example inhibit NFκB signaling through direct binding to p65 or Akt-mediated inactivation of glycogen synthase kinase (GSK)-3β. In addition, PPARβ/δ can activate adenosine monophosphate-activated protein kinase (AMPK) through phosphorylation, resulting in the inactivation of p300 and activation of SIRT1 leading to a marked reduction in acetylation of p65 inhibiting the NFκB transcriptional activity [8]. Inflammation-related target genes of PPARβ/δ are for example TGFβ, 14-3-3α, superoxide dismutase (SOD), catalase, thioredoxin, and G protein signaling-4 and -5. However, the anti-inflammatory effect of PPARβ/δ is not only mediated by the induction of anti-inflammatory genes. An example of transrepression of pro-inflammatory genes is the inhibition of the anti-inflammatory corepressor BCL-6 by inactive PPARβ/δ [8].

HIF-1α is besides NF-κB another major gene that regulates inflammation [81]. Notably, Inoue and coworkers have reported the crosstalk of the PPARβ/δ and hypoxia-inducible factor HIF-1α signaling axes in ECs upon hypoxia-induced migration [82]. During osteoblast differentiation PPARβ/δ is regulated in a HIF-1α-dependent manner [83]. In addition, PPARβ/δ regulates HIF-1α expression via calcineurin promoter binding [84]. A possible connection between PPARβ/δ and HIF-1α is intriguing as the ancient HIF-1α pathway, operating through prolyl hydroxylase domain proteins, has been identified as a central player in mouse regeneration [37]. In future studies, it will be interesting to determine if PPARβ/δ-induced/enhanced healing or regeneration is mediated through HIF-1α or can be enhanced by modulating HIF-1α activity.

### 3.4. Proliferation

Recent years have revealed that proliferating cells such as stem cells and cancer cells exhibit high levels of glycolysis while differentiated, postmitotic cells utilize fatty acid oxidation [39,85]. Thus, it is not surprising that manipulation of PPARβ/δ activity affects both proliferation and differentiation of a large variety of somatic and cancer cell types. However, it is important to note that the effect of altered PPARβ/δ activity on proliferation and differentiation is cell type- and context-dependent. For example, it has been shown that increased PPARβ/δ activity promotes proliferation of endothelial progenitor cells as well as somatic ECs [86,87,88]. In addition, it promotes proliferation of cells like cardiomyocytes [9] and hepatocellular carcinoma cell lines [89]. In contrast, ligand activation of PPARβ/δ inhibits proliferation of vascular smooth muscle cells (VSMCs) [90,91,92], HaCaT keratinocytes [93], as well as breast cancer cell lines [94] and PPARβ/δ deletion promotes cancer EC proliferation [23].

How PPARβ/δ regulates proliferation remains unclear. The analysis of the available literature reveals that mainly the up- or downregulation of classical cell cycle promoting proteins has been reported such as Cyclin A (VSMC [95]), Cyclin D1 (VSMC [91]; embryonic stem cells (ESCs) [96]; Sertoli cells [97]), Cyclin D2 (Sertoli cells [97]), Cyclin E (ESC [96]; primary thyroid cells; mouse embryonic fibroblasts [98]), cdk2 (ESC [96]; VSMC [95]), and cdk4 (VSMC [90,91]; ESC [96]). In addition, few studies have determined the effect on the cell cycle inhibitors p21 (VSMC [90]; ESC, [96]), p27 (ESC [96]; Sertoli cells [97]), p53 (VSMC [90]), and p57 (cancer ECs [23]; VSMC [95]). In addition, PPARβ/δ regulates the transcription of growth factors that promote proliferation (heparin-binding epidermal growth factor-like growth factor (HB-EGF), adult primary epidermal keratinocytes, [99]). Moreover, several pathways have been suggested to mediate the effect of PPARβ/δ on proliferation including Akt (endothelial progenitor cells (EPCs) [86]; keratinocytes [100]), p38 MAPK (ESCs [101]), extracellular signal-regulated kinase (ERK) (Sertoli cells [97]; keratinocytes [102]), and Wnt/β-catenin signaling (ESCs [101]; cardiomyocytes [9]; epithelial cells [103]).

An example of cell type-specific regulation of proliferation is the binding of PPARβ/δ to the leptin promoter, resulting in decreased leptin expression and increased liposarcoma cell proliferation [104]. In addition, it has been demonstrated that silent mating type information regulation 2 homolog 1 (sirtuin 1) mediates the anti-proliferative effect of PPARβ/δ in VSMCs [105].

### 3.5. Differentiation

In agreement with the idea that differentiation is accompanied by a switch from glycolysis to fatty acid oxidation, ESC differentiation to cardiomyocytes involves upregulation of oxidative phosphorylation and downregulation of glycolysis [106]. In contrast, reprogramming fibroblasts to induced pluripotent stem cells is dependent on induction of glycolysis [107]. A metabolic switch from glycolysis to fatty acid oxidation occurs also during differentiation of immature somites to muscle progenitors [108] and during heart development when the mode of heart growth switches from hyperplasia (proliferation) to hypertrophy (increase in cell size) [102,109]. Moreover, multiple signaling pathways affecting differentiation control also cellular metabolism such as the phosphatidylinositol-4,5-bisphosphate 3-kinase (PI3K)/AKT/mammalian target of rapamycin (mTOR), the Ras, the liver kinase B (Lkb1)/AMPK, and the Hedgehog pathways [39]. Consequently, it could also be demonstrated that alterations of PPARβ/δ activity or signaling affect differentiation. For example, PPARβ/δ controls on a transcriptional level the endothelial differentiation gene (Edg)-2 and PPARβ/δ agonist stimulation enhances the vasculogenic potential of endothelial progenitor cells (EPCs) [110]. Moreover, it has been shown that PPARβ/δ can promote osteoblast differentiation via Wnt signaling [30], in a keratinocyte fatty acid binding protein (K-FABP)-dependent manner keratinocyte differentiation [111], early adipocyte differentiation via PPARγ [112,113], late sebocyte differentiation [114], oligodendrocyte [115,116] and neural [117] differentiation, as well as p53- and SOX2-mediated differentiation of neuroblastoma cells [118]. Finally, the PPARβ/δ target gene FoxO1 plays an important role as negative regulator of skeletal muscle differentiation [119,120].

### 3.6. Angiogenesis

Tissues and organs are vascularized to provide their cells with oxygen and nutrients as well as to remove metabolic waste products. Thus, new vessels have to be formed after an injury to maintain regenerated tissue. This process is called angiogenesis or neo-angiogenesis. As described under Section 3.4, PPARβ/δ is involved in the regulation of EPC, EC and VSMC proliferation. In addition, it has been shown that PPARβ/δ activation inhibits IL1β-stimulated VSMC migration via upregulation of IL-1 receptor antagonist and was associated with the down-regulation of matrix metalloproteinase (MMP)-2 and MMP-9 [90]. Moreover, oxidized low-density lipoprotein-induced VSMC migration was inhibited in a SIRT1-dependent manner by PPARβ/δ activation [105]. That PPARβ/δ directly regulates physiological angiogenesis has been demonstrated in skeletal [121] and cardiac [84] muscle by utilizing agonists and/or transgenic mice overexpressing PPARβ/δ in skeletal muscle cells. These data showed that PPARβ/δ bound directly to the calcineurin promoter inducing the expression of its target genes such as HIF-1α. Consequently, inhibition of calcineurin activity abolished the angiogenic response to PPARβ/δ agonist stimulation [84]. Notably, the effect of PPARβ/δ transgenic overexpression on angiogenesis was significantly lower than the effect of agonist treatment [121]. In a subsequent study, it has been revealed that PPARβ/δ agonist stimulation of EPCs resulted in MMP-9 expression by direct transcriptional activation which caused insulin-like growth factor-binding protein (IGFBP) 3 degradation and thus IGF-1 release. Conditioned medium of stimulated EPCs enhanced the number and functions of human umbilical vein ECs and C2C12 myoblasts via IGF-1 receptor activation. Importantly, PPARβ/δ agonist stimulation in vivo in a mouse hind limb ischemia model induced in an MMP-9-dependent manner IGF-1 receptor phosphorylation in ECs and skeletal muscle and promoted angiogenesis and skeletal muscle regeneration [122]. In the same study, the authors report that the same pro-angiogenic mechanism can be induced by PPARβ/δ agonist stimulation in a mouse skin punch wound model.

In addition to its pro-angiogenic effects, PPARβ/δ agonists have been shown to be vasoprotective by activating and or increasing the expression of endothelial nitric oxide synthase (eNOS) [123]. Importantly, injury to the endothelium (e.g., through angioplasty) results in inefficient regeneration as the regenerated endothelium cannot produce enough nitric oxide causing local nitric oxide deficiency which can lead to intravascular coagulations, vasospasm, and inflammation-mediated atherosclerosis [124]. Thus, PPARβ/δ agonists might be useful as anti-thrombotic and anti-atherosclerotic drugs.

Besides a physiological role of PPARβ/δ in angiogenesis, evidence is accumulating that modulation of PPARβ/δ can be used to control neo-angiogenesis during pathological conditions. For example, intravitreal injection of the PPARβ/δ antagonist GSK0660 inhibited neovascularization in a rat oxygen-induced retinopathy and reduced serum-induced in human retinal microvascular ECs proliferation and tube formation. Both cases were correlated with the reduced expression of the pro-angiogenic angiopoietin like (Angptl) 4. In contrast, the agonist PPARβ/δ GW0742 increased neovascularization and tube formation as well as Angptl4 expression [28]. In addition, tumor transplantation assays as well as Matrigel plug assays utilizing PPARβ/δ knockout mice indicate that PPARβ/δ is required for the formation of functional tumor microvessels [23].

## 4. PPARβ/δ in Wound Healing and Regeneration

It is essential for species to deal with injuries to survive. Notably, almost all species have some regenerative capacity, including humans. For example, they can regenerate liver [5] and bone [125,126]. The basic steps of regeneration are: (1) an inflammatory response [72] induced by injuries caused by infection, intoxication or mechanical infliction due to signal molecules released by dead or dying cells or invading organisms [127]; (2) wound healing [128] that can be accompanied by a transient scar [129]; (3) ECM remodeling to allow migration as well as induction of proliferation with subsequent differentiation to generate new tissue [6]; (4) an anti-inflammatory and anti-fibrotic response [72]; and (5) remodeling of the tissue to achieve a functional state [6,72,128]. As described above, PPARβ/δ is involved in all these mechanisms. In recent years, it has been shown that manipulation of PPARβ/δ activity is inhibiting or promoting healing as well as regeneration of a large variety of tissues/organs.

### 4.1. Skin

The skin is the largest organ of the human body consisting of epidermis and dermis. The epidermis consists of five layers, forming a protective outer barrier. The dermis consists of connective tissue and is separated from the epidermis by a thin sheet of fibers called the basement membrane. The dermis provides tensile strength and elasticity to the skin and serves as a location for the appendages of skin such as hair follicles, nails, and sweat glands. Skin injuries in adult mammals result usually in scar tissue that lack skin appendages. As long as the deepest layer of the epidermis, the basal layer containing stem cells, is not injured the mammalian skin can heal without forming a scar. Yet, deep injuries and third degree burns fail to regenerate and result into scarring or chronic wounds. As soon as a wound exceeds 4 cm in diameter, a tissue graft is needed. Yet, while enormous progress has been made in tissue engineering due to the clinical importance, to date there is no complete functional skin substitute available (reviewed in [130]).

Similar to other organs, wound healing is initiated by inflammation, followed by reepithialization due to proliferation and migration of keratinocytes. In parallel fibroblast proliferation is activated and angiogenesis is induced. In addition, fibroblasts produce collagens and other extracellular matrix proteins to aid in wound repair (reviewed in [131]). As PPARβ/δ has multiple functions in skin health and disease such as has pro-differentiating effects on keratinocytes, PPARβ/δ appears to be an ideal therapeutic target to enhance endogenous regenerative skin regeneration capacities [24].

That PPARβ/δ is involved in skin healing has been suggested based on the finding that its expression is strongly induced upon injury by inflammatory cytokines (e.g., TNF-α, [59]) and keratinocytes at the edges of wounds maintain high expression as long as the repair process has not been completed [132]. The analysis of wound healing in PPARβ/δ knockout mice revealed that PPARβ/δ is required during skin healing for keratinocyte proliferation resulting in a delay in healing by two to three days [132]. Activated PPARβ/δ signals via the PI3K/Akt1 pathway, which mediates cell survival via inactivation of BAD (BCL2-associated agonist of cell death) and adhesion as well as migration via inhibition of GSK3β [24,133]. During progression of the healing process, PPARβ/δ expression is decreasing mediated by transforming growth factor (TGF) β1-induced Smad3/Smad4 repressor complexes. Notably, keratinocyte proliferation is also regulated by dermal fibroblasts. An injury causes IL-1 secretion by keratinocytes, which activates in fibroblasts via IL-R1 the transforming growth factor beta-activated kinase 1 (TAK) 1/cJun/AP1 pathway resulting in growth factor and cytokine release promoting keratinocyte proliferation. In fibroblasts, however, activated PPARβ/δ induces the expression of the secretory IL-1 receptor antagonist sIL-1Ra. This attenuates the IL1 responsiveness of fibroblasts resulting in decreased secretion of pro-proliferative factors and thus reduced keratinocyte proliferation [134]. This regulatory mechanism demonstrates how important the local activation of PPARβ/δ is.

The available data on PPARβ/δ in regards to wound healing but also skin disorders has recently in detail been reviewed [24,25,34]. Yet, there appear to be no studies that attempted to utilize this knowledge to significantly enhance the regenerative capacity at least in mice or rat.

### 4.2. Corneal Epithelial Wound Healing

Nakamura and coworkers found that after surgical removal of corneal epithelium PPARβ/δ expression was temporally upregulated at the wound’s edges like observed in skin wound healing. This phenomenon was additionally observed in a human corneal epithelial wound model ex vivo. PPARβ/δ activation enhanced healing of experimental corneal epithelial wounds in rats and wound closure in an in vitro system based on human corneal epithelial cells. Finally, PPARβ/δ activation was sufficient to inhibit TNFα–induced cell death of corneal epithelial cells [31]. If wound healing was impaired or the lesion was too large, activated keratocytes migrated, proliferated, and differentiated into fibroblasts and myofibroblasts leading to an altered ECM and corneal opacity. Gu and coworkers tested the effects of PPARβ/δ agonists in a model of corneal wound healing upon epithelial defects generated by laser ablation [74]. They observed that the agonist inhibited early stages of wound healing reepithelialization and promoted angiogenesis. Yet, during the remodeling phase agonist treatment decreased keratocyte transdifferentiation into myofibroblasts and thus also ECM synthesis/scaring and corneal opacity. These examples represent another good example for the need of a local and timed modulation of PPARβ/δ activity.

### 4.3. Reendothelialization

As described under Section 3, PPARβ/δ is involved in the regulation of EPC, EC, and VSMC proliferation and/or migration and can promote neo-angiogenesis. In addition, He and coworkers have shown in a mouse model of carotid artery injury that PPARβ/δ agonist treatment of human EPCs significantly enhanced the ability of transplanted EPCs to repair denuded endothelium. PPARβ/δ agonist treatment of human EPCs increased the production of tetrahydrobiopterin, an essential co-factor of eNOS, as well as expression and activity of GTP cyclohydrolase I, the rate-limiting enzyme responsible for de novo synthesis of tetrahydrobiopterin. These effects were dependent on PPARβ/δ agonist-induced suppression of the phosphatase and tensin homolog (PTEN) expression thereby promoting AKT signaling. Notably, PPARβ/δ agonist-induced EPC proliferation was primarily dependent on BH4 but independent of NO, while induced EPC migration was dependent on both [88].

### 4.4. Skeletal Muscle

Regeneration of skeletal muscle is among the best-understood regenerative processes in mammalians, including humans. Similar to bone, mammalian skeletal muscle can regenerate but the extent of regeneration is limited [135,136]. If an injury exceeds the endogenous regenerative capacity the skeletal muscle scars. This kind of injury does occur not only after an accident but are often also caused by surgical interventions such as total hip or knee arthroplasty [136]. Moreover, skeletal muscle loss occurs in a variety of congenital diseases (myofibrillar myopathies, [137]), cancer (cachexia, [138]), as well as aging (sarcopenia, [139]). As maintaining skeletal muscle function is essential for good health and independent living, there is a great interest in developing strategies to enhance the endogenous regenerative capacity of skeletal muscle or to generate muscle by stem cell-based therapies.

The mechanism of skeletal muscle regeneration has recently been reviewed in detail [135,136]. Briefly, the main cell type during skeletal muscle regeneration is the resident muscle stem (satellite) cell (MuSC). After an injury MuSCs are activated, enter the cell cycle, proliferate, differentiate into myoblasts, which finally fuse to damaged fibers or generate myofibers de novo. Maintenance of MuSCs is for example dependent on paired box proteinPax7, FoxO1/Notch signaling, and the ECM via β1-integrin signaling [119,135,140]. Yet, skeletal muscle regeneration is a complex process that involves several cell types (e.g., immune cells, adipogenic progenitors, fibroblasts, and pericytes) that interact with each other. The basic steps in skeletal muscle regeneration are: (1) bleeding triggering coagulation and hematoma formation; (2) induction of a pro-inflammatory reaction (e.g., MuSC-mediated recruitment of immune cells such as pro-inflammatory M1 macrophages); (3) induction of MuSC proliferation (e.g., by IL-6 secreted by M1 macrophages); (4) switching of immune cells such as macrophages to anaerobic glycolysis due to a hypoxic environment; (5) appearance of anti-inflammatory M2 macrophages, which inhibit myoblast proliferation and stimulate the subsequent differentiation and fusion of myofibers; and (6) initiation of re-vascularization. Notably, the cytokine pattern and mechanical tension decides, during the initial inflammatory reaction, whether fibroblasts differentiate into myofibroblasts promoting fibrosis or whether regeneration will occur [70].

The role of PPARβ/δ in skeletal muscle physiology and pathophysiology has recently been reviewed by Manickham and Wahli [21]. In 2009, Giordano and coworkers demonstrated that muscle-specific overexpression of PPARβ/δ as well as pharmacological activation promotes skeletal muscle fusion but not proliferation of MuSCs [141]. This is in agreement with the recent finding by Lee and coworkers showing that PPARβ/δ agonist treatment, as well as PPARβ/δ overexpression, enhanced C2C12 myotube formation via p38 MAPK and Akt [142]. Conditional PPARβ/δ knockout mice utilizing Myf5-Cre deleter lines (affect MuSCs) exhibited no gross morphological phenotype. A detailed analysis revealed a reduced number of MuSCs (~40%) and a delayed regenerative response to cardiotoxin-induced injury. The number of small regenerating fibers was increased by ~30% while the number of large regenerating fibers was decreased by 20%. Moreover, MuSCs from conditional PPARβ/δ knockout mice displayed reduced in vitro and in vivo MuSCs proliferation but enhanced differentiation. These phenomena were associated with a downregulation of FoxO1, which is a negative regulator of skeletal muscle differentiation [120]. In 2015, Chandrashekar and coworkers reported that PPARβ/δ knockout mice exhibit reduced skeletal muscle weight and myofiber atrophy during postnatal development [143]. In agreement with the conditional knockout mice, the number of MuSCs was reduced (~25%). Yet, mass was affected in PPARβ/δ knockout mice while myofiber number was not significantly altered. Moreover, PPARβ/δ knockout mice contained significantly less myoblasts upon notexin-mediated injury (~50% at 28 dpi). In addition, the authors observed in the knockout animals increased necrosis (three days post injury (dpi)) and myofibers containing centrally located nuclei were smaller (7 dpi). While previous PPARβ/δ-related studies investigated mainly the effect on MuSCs, Chandrashekar and coworkers describe an increased infiltration of macrophages at 3 dpi. Yet, loss of PPARβ/δ did not significantly alter scar tissue formation or metabolic properties of regenerated muscle.

Even though several groups describe modulation of PPARβ/δ as affecting MuSCs as well as immune cells, the data obtained from mouse models utilizing overexpression and knockout strategies do not clarify if skeletal muscle regeneration can be enhanced by altering PPARβ/δ activity. In contrast, Haralampieva and coworkers aimed at manipulating MuSCs directly in order to enhance their regenerative capacity. For this purpose, they have overexpressed human peroxisome proliferator-activated receptor gamma coactivator 1-alpha (hPGC-1α) in hMuSCs and tested the effect in a crush-induced injury model [144]. The authors observed a decreased inflammatory response accompanied by enhanced expression of muscle markers in newly formed myotubes and increased muscle contraction force. Thus, injected hMuSCs overexpressing PGC-1α enhanced functional muscle regeneration after injury.

Collectively, these data indicate that PPARβ/δ is involved in MuSC proliferation but also in myoblast fusion [119]. It remains elusive if modulation of PPARβ/δ can be utilized to enhance regeneration even though it is involved in a large number of processes affecting skeletal muscle regeneration.

### 4.5. Bone

The bone is one of the few tissues/organs of the human body that can heal and regenerate [125,126]. Yet, the regenerative capacity is limited, does not occur in all cases and regeneration is complicated by comorbidities such as type 2 diabetes. That PPARβ/δ might play a role in bone regeneration has been suggested by the bone phenotype of PPARβ/δ knockout mice. These mice were characterized by increased myostatin expression, low bone formation, and increased resorption resulting in decreasing bone strength with age. In addition, they did not respond with bone formation upon exercise [145]. Conditional knockout mice utilizing a SOX2-Cre deleter line showed substantial osteopenia paralleled by lower serum concentrations of osteoprotegerin and osteocalcin, a higher RANKL-to-osteoprotegerin ratio, as well as a higher number of osteoclasts within the trabecular bones [30]. In contrast, activation of PPARβ/δ in vitro promoted osteogenic differentiation of osteoblasts and inhibited in co-cultures of osteoblasts and osteoclasts osteoclast differentiation and bone resorption. Moreover, pharmacological activation of PPARβ/δ in a mouse model of postmenopausal osteoporosis led to normalization of the altered RANKL-to-osteoprotegerin ratio and the restoration of normal bone density [30].

### 4.6. Liver

The mammalian liver can regenerate based on hepatocyte proliferation in contrast to skin, skeletal muscle, and bone after large injuries such as two-thirds partial hepatectomy [5]. Liu and coworkers demonstrated, utilizing PPARβ/δ knockout mice, that PPARβ/δ is required for the activation of hepatocyte proliferation upon injury to enable liver regeneration. A detailed analysis of their model revealed that PPARβ/δ deficiency blocked the induction of genes involved in glycolysis, the activation the PDK1/AKT pathway at 36 to 48 h after injury, as well as the proliferation associated transcription factors E2F1, 2, 7, and 8 resulting in delayed liver regeneration [33].

### 4.7. Cardiac Muscle

Significant effort is invested to develop novel regenerative therapies for the injured mammalian heart as heart failure represents a major socioeconomic burden [146]. Due to the fact that the embryonic heart growth during development is mediated by cardiomyocyte proliferation and as natural occurring heart regeneration in zebrafish and newt is based on the same cellular mechanism, one possible future approach appears to be the induction of adult mammalian cardiomyocyte proliferation [147]. Yet, it is poorly understood why mammalian cardiomyocytes stop proliferating shortly after birth. Recently, Magadum and coworkers wondered whether the metabolic shift in cardiomyocytes around birth from glycolysis to fatty acid oxidation to ensure ATP generation might be responsible for this phenomenon [9]. In the adult heart, about 70% of the cardiac energy metabolism relies on the oxidation of fatty acids and 30% on glucose, lactate, and ketone bodies. Notably, the heart can in contrast to other organs adapt its energy metabolism based on substrate availability [102,148]. Activation of PPARβ/δ in neonatal cardiomyocytes induced their proliferation via the PDK1/p308Akt/GSK3β/β-catenin pathway. This proliferative response could even be further enhanced by treatment with the GSK3β inhibitor 6-bromoindirubin-3′-oxime (BIO). Moreover, inhibition of PPARβ/δ reduced cardiomyocyte proliferation during zebrafish heart regeneration. Finally, genetic as well as pharmacological activation of PPARβ/δ in a myocardial infarct model induced cell cycle progression in cardiomyocytes, reduced scarring, and improved cardiac function [9]. While it has not been proven to what extent cardiomyocyte proliferation upon PPARβ/δ activation contributes to improved function, it appears likely that it is due to the pleiotropic effects of PPARβ/δ: (1) inhibiting apoptosis (see Section 3.2 and [54]); (2) modulating inflammation and inhibiting fibrosis (Section 3.3); (3) promoting cardiomyocyte proliferation (Section 3.4); (4) promoting angiogenesis (Section 3.6).

In addition to its role in healing and regeneration, PPARβ/δ-mediates, as recently reviewed, healing of metabolic diseases such as diabetes [11,149] and tissue protection [54,150,151,152,153,154].

## 5. Conclusions

**Figure 1:**
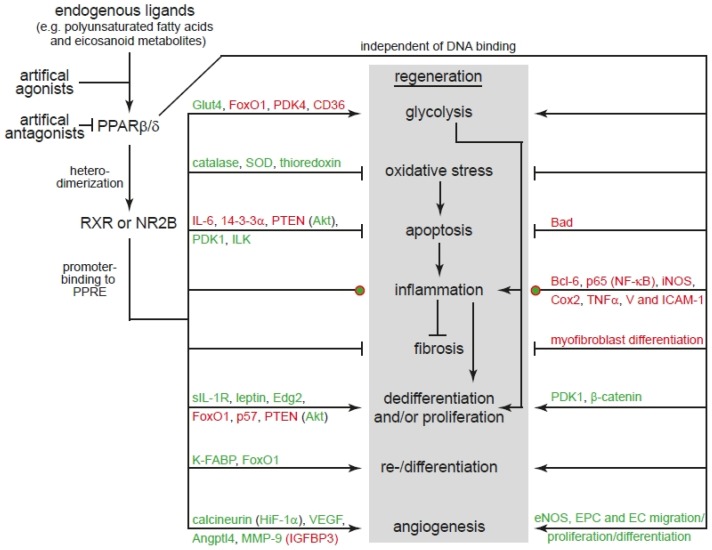
Examples of direct targets of PPARβ/δ involved in the different processes of regeneration. Red: inactivation. Green: activation. Affected pathways are indicated in brackets. Note: inflammation needs first to be activated and later inhibited during regeneration.

Our literature analysis confirms that modulation of PPARβ/δ activity can regulate all cellular processes of regeneration (see Section 3 and Figure 1). However, it is important to consider that for example PPARβ/δ activation can have different outcomes not only in different but also the same cell type depending on intracellular and extracellular conditions (see Section 3). Importantly, our analysis also reveals that PPARβ/δ is involved in natural occurring regeneration of mammalian organs (Figure 1). However, very little information is available on the role of PPARβ/δ in model organisms characterized by extensive regenerative capacities such as zebrafish, the ability of enhancing natural occurring but limited regeneration (e.g., liver, bone, skin; see Section 4), and to induce regeneration of mammalian organs that have no significant endogenous regenerative capacity such as the brain and the heart. Our analysis also shows that the main signaling pathways controlled by PPARβ/δ have been demonstrated to be essential for regeneration (Figure 1). For example, Akt signaling is well known to be required for mammalian liver [155], skeletal muscle [156,157] and hair follicle [158], as well as planarian [159] regeneration and its activation promotes axonal regeneration in mice [160,161,162]. In addition, it has been shown that GSK3β or β-catenin are involved in regeneration of for example mature pancreatic acinar cells [163] and intestine [164] in mice; limb regeneration in the model organisms *axolotl*, *Xenopus*, and *zebrafish* [165]; and *zebrafish* heart regeneration [166]. Furthermore, GSK3β/β-catenin signaling also enhances skeletal muscle [167] and bone [168] regeneration and can induce mammalian cardiomyocyte proliferation [169] and central nervous system axon regeneration [170]. Finally, the class of FoxO transcription factors have been shown to play a role in stem cell aging [171]. Reduced FoxO1 expression accelerates skin wound healing [172] and skeletal muscle regeneration [173]. Moreover, it inhibits axon regeneration in *C. elegans* [174]. FoxO3 is known to inhibit oligodendrocyte progenitor cell and thus myelination [175]. In addition, it plays a role in mammalian spinal cord [176], skeletal muscle [177], and liver [178] regeneration. Collectively, modulation of PPARβ/δ has a great therapeutic potential to enhance or even promote regeneration and thus we suggest to intensify the analysis of PPARβ/δ signaling in regenerative model organisms in comparison to mammals.

## Abbreviations

AF	Activation function
AMPK	Adenosine monophosphate-activated protein kinase
Angptl4	Angiopoietin like 4
ATP	Adenosine triphosphate
BAD	BCL2-associated agonist of cell death
BCL-6	B-cell lymphoma 6
CD36	Cluster of differentiation 36
COX2	Cyclooxygenase 2
CRABP-II	Cellular retinoic acid binding protein-II
DBD	DNA-binding domain
EC	Endothelial cell
ECM	Extracellular matrix
Edg	Endothelial differentiation gene
eNOS	Endothelial nitric oxide synthase
EPC	Endothelial progenitor cell
ESC	Embryonic stem cell
FABP	Fatty acid binding protein
FoxO1	Forkhead box protein O1
Glut4	Glucose transporter type 4
GSK-3β	Glycogen synthase kinase 3β
GTP	Guanosine triphosphate
HB-EGF	Heparin-binding epidermal growth factor-like growth factor
HIF-1α	Hypoxia-inducible factor-1α
hPGC-1α	Human peroxisome proliferator-activated receptor gamma coactivator-1α
IGF-1	Insulin-like growth factor 1
IGFBP	Insulin-like growth factor-binding protein
IL-1	Interleukin
iNOS	Inducible nitric oxide synthase
K-FABP	Keratinocyte-fatty acid binding protein
LBD	Ligand-binding domain
LKB1	Liver kinase B1
MAPK	Mitogen-activated protein kinase
MMP	Matrix metalloproteinase
MRL	Murphy Roths Large
MuSC	Muscle stem (satellite) cell
mTOR	Mammalian target of rapamycin
NCOR1	Nuclear receptor corepressor 1
NF-κB	Nuclear factor κ-light-chain-enhancer of activated B cells
PDK4	Pyruvate dehydrogenase kinase 4
PGC1α	PPAR coactivator 1α
PI3K	Phosphatidylinositol-4,5-bisphosphate 3-kinase
PPAR	Peroxisome proliferator-activated receptor
PPRE	Putative PPAR response element
PTEN	Phosphatase and tensin homolog
RA	Retinoic acid
RANKL	Receptor activator of nuclear factor kappa-Β ligand
RXR	Retinoic acid receptor
SIRT1	Silent mating type information regulation 2 homolog 1
SOD	Superoxide dismutase
SOX2	SRY (sex determining region Y)-box 2
TGFβ	Transforming growth factor β
TNFα	Tumor necrosis factor α
TRPV1	Transient receptor potential vanilloid type 1
VEGF	Vascular endothelial growth factor
VSMC	Vascular smooth muscle cells
